# Landfill Leachate from an Urban Solid Waste Storage System Produces Genotoxicity and Cytotoxicity in Pre-Adolescent and Young Adults Rats

**DOI:** 10.3390/ijerph182111029

**Published:** 2021-10-20

**Authors:** Omar Ricardo Torres-González, Ivan Moisés Sánchez-Hernández, Mario Eduardo Flores-Soto, Verónica Chaparro-Huerta, Cesar Soria-Fregozo, Laura Hernández-García, Eduardo Padilla-Camberos, José Miguel Flores-Fernández

**Affiliations:** 1 Unit of Medical and Pharmaceutical Biotechnology, Center for Research and Assistance in Technology and Design of the State of Jalisco, A.C. (CIATEJ), Guadalajara 44270, Mexico; polimerasados@gmail.com (O.R.T.-G.); isanchez@ciatej.mx (I.M.S.-H.); 2 Laboratorio de Neurobiología Celular y Molecular, División de Neurociencias, Centro de Investigación Biomédica de Occidente (CIBO), Instituto Mexicano del Seguro Social, Guadalajara 44340, Mexico; mario.flores@hotmail.es (M.E.F.-S.); veronicach73@gmail.com (V.C.-H.); 3 Laboratorio Ciencias Biomédicas, Departamento de Ciencias de la Tierra y de la Vida, Centro Universitario de los Lagos, Universidad de Guadalajara, Lagos de Moreno 47460, Mexico; csoria@culagos.udg.mx; 4 Department of Research and Innovation, Universidad Tecnológica de Oriental, Oriental 75020, Mexico; laura.hernandez@utdeoriental.edu.mx; 5 Department of Biochemistry & Centre for Prions and Protein Folding Diseases, University of Alberta, 204 Brain and Aging Research Building, Edmonton, AB T6G 2M8, Canada

**Keywords:** leachate, urban solid waste storage system, genotoxicity, cytotoxicity

## Abstract

Landfill leachate is a complex mixture of organic and inorganic molecules, as well as environmental pollutants that can cause harm to ecosystems and living beings. The micronucleus test in peripheral blood erythrocytes was used to evaluate the genotoxic and cytotoxic effects of exposure to a landfill leachate from an outdoor solid waste storage system on Wistar strain rats at different developmental stages, pre-adolescents and young adults, and the heavy metal content of the leachate was determined by atomic absorption spectrometry. Contents of arsenic, cadmium, chromium, mercury, and lead in the landfill leachate were outside the allowable international standards, and the exposure to the landfill leachate caused genotoxic and cytotoxic effects on Wistar rats, where the pre-adolescent animals were more susceptible to the toxics contained in the landfill leachate than young adults. Heavy metals contained in landfill leachate, individually or synergically with other molecules can be responsible for clastogenic and cytotoxic effects that can be harmful to humans and ecosystems.

## 1. Introduction

Urban solid waste storage systems (USWSSs), such as the open-air landfill, generate leachates that pollute the air, soil, and water, which threatens the health of ecosystems and living beings [1]. These landfill leachates are formed as a result of solutions percolating through trash and undergoing a biochemical transformation process. In addition, this mixture contains considerable concentrations of heavy metals and substances classified as pollutants of worldwide concern [2]. Toxicogenetic effects and consequences of landfill leachate exposure on microbes and plants as well as birds and fish are well-documented [1,3]. Nevertheless, the study of genotoxic and cytotoxic effects exclusively in terrestrial species, such as mammals, is limited [1,4,5,6,7,8,9]. Therefore, in this study, the genotoxic and cytotoxic effects of exposure to a landfill leachate from an outdoor solid waste storage system on Wistar strain rats during their pre-adolescent and young adulthood developmental stages were evaluated.

## 2. Materials and Methods

### 2.1. Landfill Leachate Sample Collection

An instantaneous sampling of the leachate from an outdoor USWSS located in the city of Guadalajara, Jalisco, Mexico was carried out. The sample was transported to the laboratory in sterile 1 L glass containers. The leachate was filtered with Whatman^®^ (Maidstone, UK) 0.2 µM to remove any suspended solid impurity.

### 2.2. Preparation of Landfill Leachate

Leachate was centrifuged at 3000× *g* for 10 min; the supernatant was considered as the 100% stock sample and stored at 4 °C until use. For the biological test, two concentrations of the leachate (LCH5%: 5% *v/v*, and LCH25%: 25% *v/v*) were prepared in distilled water.

### 2.3. Heavy Metal Analysis

The concentrations of heavy metals, arsenic (As), cadmium (Cd), chromium (Cr), mercury (Hg), and lead (Pb), were determined using the atomic absorption spectroscopy technique. Measurements were carried out at the Analytical and Metrological Services Unit of the Center for Research and Assistance in Technology and Design of the State of Jalisco.

### 2.4. Animals

A total of 60 male Wistar rats were provided by the Centro de Investigación Biomédica de Occidente (CIBO), Instituto Mexicano del Seguro Social (IMSS). The animals were housed in translucent acrylic boxes (44 × 33 × 25 cm) placed in a living room at room temperature, with a 12 × 12 h light/dark cycle (light was turned on at 7:00 am) and ad libitum access to water and food. Animals were handled following the animal care guidelines by Federal Government of Mexico (NOM-062-ZOO-1999) and the study was approved by the Committee for Ethics and Health Research of CIBO-IMSS with registration number R-2019-1305-6.

### 2.5. Animal Experiment Design

The experimental design involved three developmental stages in rats. For each stage of the experiment, four groups of five animals were used in each group. At the beginning of the experiments, pre-adolescent rats were 30 days old, whereas young adults were 60 and 90 days old for the second and third stages [10]. Five percent landfill leachate was administered to the first group (LCH5%) and 25% to the second group (LCH25%), while 20 mg/kg cyclophosphamide was administered to the third group (positive control), and distilled water was given to the control group (negative control). In total, 500 uL of each treatment were administered through the intragastric route with a stainless-steel cannula for 30 consecutive days. After the treatment was completed, blood was drawn from the tail vein of each animal (each group at that time was 60, 90, and 120 days old). An intraperitoneal administration of cyclophosphamide was performed 48 h prior to collecting the blood sample for the positive control (blood collection was performed at 60, 90, and 120 days old as well).

### 2.6. Genotoxicity and Cytotoxicity Test

The genotoxic and cytotoxic effect was evaluated by quantification on the erythrocyte micronucleus formation [11,12]. Peripheral blood drops were placed on prestained slides with acridine orange and the micronucleated cells were scored by a fluorescence microscope (model CX-40, Olympus, Hamburg, Germany) under a fluorescence illumination system (DMB-2 Olympus blue filter).

In total, 2000 polychromatic erythrocytes (PEs) were analyzed and the frequencies of micronucleated polychromatic erythrocytes (MNPEs) were scored on three slides per animal; the genotoxic effect was expressed as MNPE/PE. In order to assess the cytotoxic effect, the number of PE per total erythrocytes (TEs) as well on three slides from each animal was counted; cytotoxic effect was expressed as PE/TE.

### 2.7. Statistical Analysis

All data were expressed as mean ± standard error on triplicate. Genotoxicity and cytotoxicity data were analyzed by one-way ANOVA followed by Dunnett’s post hoc test to find groups with significant differences using GraphPad Prism 8.0.1 software (GraphPad Software, San Diego, CA, USA). *p* < 0.05 was considered significant.

## 3. Results and Discussion

Leachate from the sanitary landfill from the open-air storage of solid waste is a mixture of organic compounds, inorganic macromolecules, and heavy metals. The mercury level found in the landfill leachate analyzed in this study was five times higher than those allowed by the Mexican standard and 50 times above international standards (Table 1). Other heavy metals, such as arsenic, cadmium, chromium, and lead, were found to be within the Mexican standard. Nevertheless, arsenic and cadmium were found to be 10 and 50 times above the permissible limits of the international standard, as well as for chromium and lead, which were found to be 10 and >65 five times above the allowable limits (Table 1).

As a primary source of toxic substances emitted by landfills, leachates could be the main source. Heavy metals as pollutants can be found in low concentrations, but ill effects have been reported for organisms exposed to these trace quantities [1,4,6,7,14]. According to the United States Environmental Protection Agency, chromium causes allergic dermatitis and is carcinogenic, while arsenic damages skin, causes circulatory problems [15], and has neurotoxic effects, affecting speech, visual perception, and cognitive functioning [16].

Cadmium damages the respiratory system and causes neurodegenerative diseases, including Parkinson’s and Alzheimer’s diseases [17]. Cd accumulated in the renal cortex leads to renal damage [18] and contributes to bone demineralization, diabetes, and hypertension [19,20], and is carcinogenic as well.

The adverse effects of mercury exposure during gestation include neural tube defects, cleft palate, eye abnormalities, as well as neurobehavioral changes and classic birth defects, such as mental retardation, cerebral palsy, deafness, blindness, and dysarthria [21]. Moreover, Hg impairs sensory, motor, and cognitive functions; reduces kidney function [22]; and can increase the β-amyloid proteins that are associated with Parkinson’s and Alzheimer’s diseases [23,24].

The presence of lead affects intellectual functioning, memory, and visual perception abilities [25,26]; causes hemolysis, anemia, and kidney damage; reduces sperm production; and reduces vigor and libido [15]. The toxic effects of exposure to Pb among females have also been associated with miscarriages and stillbirths [27].

The genotoxicity of landfill leachate is generally measured by exposing the substance to living organisms or cells in vitro and phenotypic damage is evaluated [2]. However, sometimes, phenotypic changes or signs of toxicity cannot be easily detected, thus the micronucleus technique is an excellent biomarker to evaluate genotoxicity, recommended internationally [11] as part of safety assessment, as the acridine orange staining allows the differentiation of micronucleated polychromatic erythrocytes from polychromatic erythrocytes, indicating chromosome damage was induced.

**Figure 1:**
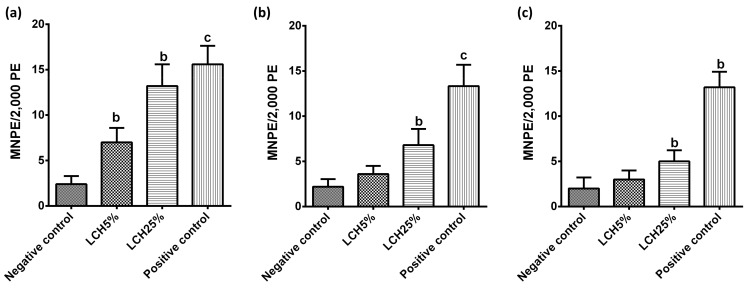
Genotoxicity effect by 30 exposure consecutive days of landfill leachate at 5% and 25% to (**a**) 60-day old pre-adolescent Wistar rats, (**b**) 90-day-old and (**c**) 120-day-old young adult rats at the end of the exposure. Micronucleated polychromatic erythrocytes per 2000 polychromatic erythrocytes (MNPE/2000 PE) in peripheral blood are represented as mean ± SE. The superscripts are significantly ((b) *p* < 0.01; (c) *p* < 0.001) different from the negative control group (distilled water). Positive control: cyclophosphamide (20 mg/kg).

After exposure to landfill leachate, pre-adolescents and 90- and 120-day-old young adults Wistar rats showed a significant increase of micronucleated erythrocytes in peripheral blood (Figure 1). The MNPE incidence induced by landfill leachate at 5% and 25% in 60-day-old pre-adolescent rats was 2.92- and 5.50-fold higher (*p* < 0.01) than the control (Figure 1a). Interestingly, landfill leachate at 5% did not show significant differences when compared to the negative control in 90- and 120-day-old young adults Wistar rats; however, landfill leachate at 25% showed significant differences (*p* < 0.01) by 3.09- and 2.5-fold compared to the negative control at this developmental stage (Figure 1b,c).

**Figure 2:**
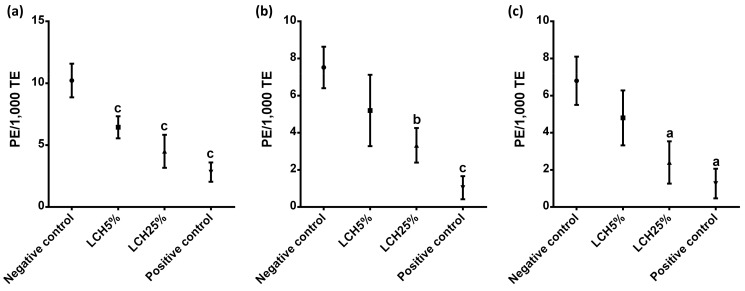
Cytotoxic effect as a result of 30 consecutive days of landfill leachate exposure at 5% and 20% to (**a**) 60-day-old pre-adolescent Wistar rats, (**b**) 90-day-old, and (**c**) 120-day-old young adult rats when the experiment concluded. Polychromatic erythrocytes per 1000 total erythrocytes (PE/1000 TE) in peripheral blood are represented as mean ± SE. The superscripts are significantly ((a) *p* < 0.05; (b) *p* < 0.01; (c) *p* < 0.001) different from the negative control. Positive control: cyclophosphamide (20 mg/kg).

A cytotoxic effect is associated with a smaller proportion of polychromatic erythrocytes per total erythrocytes. A statistically significant cytotoxic effect of landfill leachate was observed in Wistar rats of different developmental stages (Figure 2). A cytotoxic effect was observed in pre-adolescent rats (*p* < 0.001; Figure 2a), but none of these effects were observed in young adult rats of 90 or 120 days of age using landfill leachate at 5%, while 25% landfill leachate had a cytotoxic effect on both pre-adolescent and young adult rats (Figure 2).

The significant increase in the formation of MNPE among PE in the peripheral blood of the animals at different developmental stages exposed to the landfill leachate demonstrated and confirmed the clastogenic effects of the environmental contaminants present in the open-air storage of solid waste (Figure 1). Furthermore, the significant decrease of the ratio of PE to total erythrocytes in landfill leachate-treated rats (Figure 2) suggests a toxicological effect on normal bone marrow cell proliferation induced by the constituents of the leachate [6].

This study provides important and useful information on adverse effects of exposure to landfill leachates on mammalian rodents at developmental stages of pre-adolescent and young adulthood, which has not had been studied previously. Throughout history, it has been debated whether environmental pollutants influence mammalian reproduction in the later stages more than early stages. Because certain stages of development are more critical, it is very difficult to evaluate the possible effects of an exposure to a metal in terms of the chemical form, the dose, or the administration route [28,29]. Notwithstanding, the results of this study demonstrate that the LCH25% group that was administered a 5 times higher dose of landfill leachate than the LCH5% group, which therefore contained a quantity of metals five times greater, presented a higher number of micronucleated polychromatic erythrocytes and a lower number of polychromatic erythrocytes, which indicates greater genotoxicity and cytotoxicity (Figure 1 and Figure 2).

The fact that immature animals, such as is the case in this study for pre-adolescent Wistar rats, have greater absorption processes and more vulnerable organs makes them more susceptible to the effects of toxic metals and other pollutants. Senescent or aging animals are also more susceptible to genotoxic and neurotoxic contaminants, which can cause negative effects on their organs and systems.

Further studies are needed to determine the phenotypic damage and potential effects on the immune and endocrine systems.

## 4. Conclusions

The approach used to test genotoxicity and cytotoxicity in this investigation demonstrates a valuable method for determining the toxic effects of exposure to landfill leachates. Heavy metals as pollutants, outside of the allowed international limits contained in landfill leachate, could individually or synergically with other molecules be responsible for the genotoxic and cytotoxic effects in pre-adolescent and young adult Wistar rats exposed to the landfill leachate from an outdoor solid waste storage system, with animals in an earlier stage of development being more susceptible to toxic molecules.

## Figures and Tables

**Table 1:** Heavy metal content in landfill leachate and maximum permissible limits (mg/L).

Metal	Leachate *	NOM-002 **	EPA ***
As	0.10 ± 0.010	1.00	0.010
Cd	0.25 ± 0.017	1.00	0.005
Cr	1.00 ± 0.100	1.00	0.100
Hg	0.10 ± 0.005	0.02	0.002
Pb	1.00 ± 0.095	2.00	0.015

* Values were expressed as mean ± SE of triplicate analyses. ** NOM: Mexican Official Standards, NOM-002-SEMARNAT-1996 [13]. *** EPA: United State Environmental Protection Agency (https://www.epa.gov/ground-water-and-drinking-water/national-primary-drinking-water-regulations#Inorganic (accessed on 23 August 2021)).

## Data Availability

The data underlying this article will be shared on reasonable request from the corresponding author.
